# Supplementary material for: Anthropogenic fertilization influences a shift in barley rhizosphere microbial communities
Source: PeerJ. 2024 Jul 10;12:e17303. doi: 10.7717/peerj.17303 (PMC11246026; doi:10.7717/peerj.17303)
Supplement: Supplemental Information 1 [file peerj-12-17303-s001.docx]

**Supplementary Materials**

**Anthropogenic fertilization influences a shift in barley rhizosphere microbial communities**

**Enagbonma, Ben Jesuorsemwen; Fadiji, Ayomide Emmanuel and** **Babalola, Olubukola Oluranti ^⃰^**

Food Security and Safety Focus Area, Faculty of Natural and Agricultural Sciences, North-West University, Private Bag X2046, Mmabatho 2735, South Africa.

^⃰^ Correspondence: [olubukola.babalola@nwu.ac.za](mailto:olubukola.babalola@nwu.ac.za); +27183892568

**Table S1: Soil analysis before fertilization and planting of barley**

| **Physical and chemical properties** | **Values** |
| --- | --- |
| pH | 6.14 |
| Organic carbon (%) | 1.48 |
| Total nitrogen (%) | 1.71 |
| Phosphorus (mg/L) | 31.38 |
| Calcium (mg/L) | 0.27 |
| Potassium (mg/L) | 5.57 |
| Magnesium (mg/L) | 0.89 |
| Sand (%) | 94.33 |
| Silt (%) | 1.88 |
| Clay (%) | 3.63 |

**Table S2: List of MG-RAST sample access numbers/IDs**

| **Samples** | **MG-RAST ID** |
| --- | --- |
| CB1 | 03f08b5f546d676d343935363637392e33 |
| CB2 | 067f8dca0d6d676d343935363638312e33 |
| CB3 | 07e4283e626d676d343935363637382e33 |
| CB4 | 08520886d86d676d343935363636382e33 |
| CB5 | 11c734ba1a6d676d343935363636342e33 |
| CB6 | 1aaa8d70ed6d676d343935363636392e33 |
| CB7 | 21e92313db6d676d343935363636322e33 |
| CB8 | 29b90316776d676d343935363636332e33 |
| OB1 | 29bbf2c92f6d676d343935363638342e33 |
| OB2 | 2ceff91c0c6d676d343935363636372e33 |
| OB3 | 486267706f6d676d343935363637342e33 |
| OB4 | 50aca7221f6d676d343935363637352e33 |
| OB5 | 5713cfa68b6d676d343935363637332e33 |
| OB6 | 77f88e8d096d676d343935363637372e33 |
| OB7 | 91a40fdd286d676d343935363637322e33 |
| OB6 | 9f4a49bf1d6d676d343935363637312e33 |
| NB1 | a30f1fba4e6d676d343935363638322e33 |
| NB2 | c21e45b37c6d676d343935363636362e33 |
| NB3 | c9353e377f6d676d343935363636352e33 |
| NB4 | cc9f0ccc116d676d343935363637362e33 |
| NB5 | d8680414f26d676d343935363638302e33 |
| NB6 | dc3d8213c36d676d343935363638332e33 |
| NB7 | f1af224b876d676d343935363637302e33 |
| NB8 | f1f734a8ae6d676d343935363638352e33 |

**Table S3: Mean sequence information before and after pre-processes**

| **Sequence information** | **CB** | **OB** | **NB** |
| --- | --- | --- | --- |
| Upload: bp Count | 158,370,024 | 144,992,330 | 130,563,634 |
| Upload: Sequences Count (bp) | 542311.375 | 498358.875 | 446983.25 |
| Upload: Mean Sequence Length | 292 ± 4 bp | 292 ± 3 bp | 292 ± 2 bp |
| Upload: Mean GC percent | 57 ± 3 % | 57 ± 3 % | 57 ± 3 % |
| Artificial Duplicate Reads: Sequence Count | 508,191 | 467,106 | 416,206 |
| Post QC: bp Count | 9,949,276 | 8,938,469 | 8,985,746 |
| Post QC: Sequences Count | 34,107 | 30,927 | 30,773 |
| Post QC: Mean Sequence Length | 292 ± 7 bp | 292 ± 7 bp | 292 ± 5 bp |
| Post QC: Mean GC percent | 57 ± 3 % | 57 ± 3 % | 57 ± 3 % |
| Processed: Predicted Protein Features | 164 | 141 | 169 |
| Processed: Predicted rRNA Features | 102,719 | 89,267 | 103,611 |
| Alignment: Identified Protein Features | 58 | 50 | 60 |
| Alignment: Identified rRNA Features | 93,506 | 81,628 | 93,931 |

Values presented in the table are the means of the eight replicates from each fertilization site. CB = barley rhizosphere under chemical fertilization, OB = barley rhizosphere under organic fertilization, NB = bulk soil


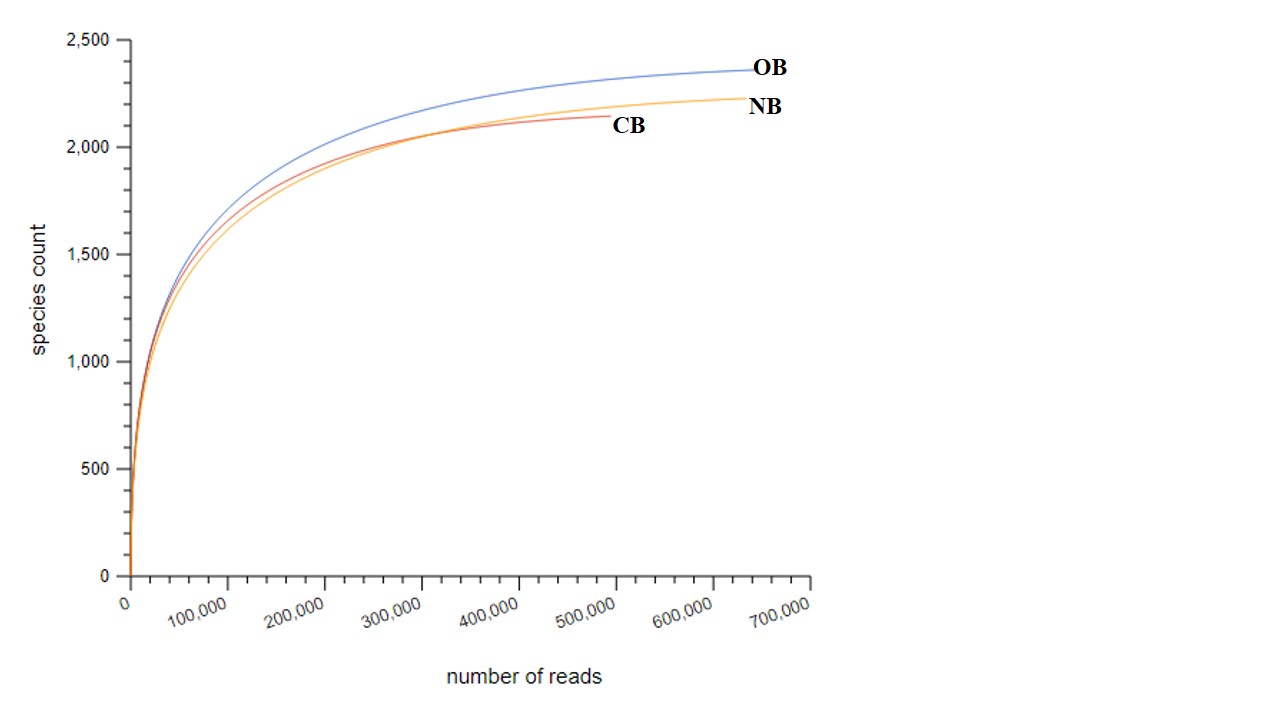


Fig S1. Rarefaction curve of species richness for barley rhizosphere bacteria. Values presented in the curve are the means of the eight replicates from each fertilization site. CB = barley rhizosphere under chemical fertilization, OB = barley rhizosphere under organic fertilization, NB = bulk soil
